# Age of Acquisition of Personality Terms: Implications for Personality Theory

**DOI:** 10.5964/ejop.2987

**Published:** 2022-05-31

**Authors:** Eka Roivainen

**Affiliations:** 1 Department of Medical Rehabilitation, Oulu University Hospital, Oulu, Finland; Maria Grzegorzewska University, Warsaw, Poland

**Keywords:** age of acquisition, personality terms, trait taxonomy, language development, personality facets

## Abstract

Analysis of the age of acquisition (AoA) of personality terms represents a genetic method for the study of the individual personality lexicon and offers a potential alternative to correlational analysis for identifying the fundamental personality descriptors among the thousands of terms that appear in language. In the present study, the relationship between AoA, word frequency, word desirability, and factor loading in the Big Five and Hexaco models of 274 and 408 personality adjectives was analyzed. It was found that young children (2nd graders or younger) acquire personality terms that represent traits at the core of the broad personality factors in the Big Five and Hexaco models slightly earlier than words that represent more peripheral traits. In older children beyond second grade, the correlation between factor loading and AoA is weak. Words that describe the broad openness and stability/emotionality aspects of personality are learned later than words for the other broad factors. Word frequency (in book texts) and desirability have a weak negative correlation with AoA. It is hypothesized that the AoA of a personality term reflects the importance of the corresponding trait for children and may be used as one criterion for ranking facet level traits independent of the broad factors.

Prevailing trait taxonomies in contemporary personality science are based on the factor analysis of personality descriptions. McCrae and Costa (1987) analyzed a large pool of personality test items (short statements describing behavior or attitudes) and found that five broad personality factors underlie the multitude of test items. The five broad factors in McCrae and Costa’s Five-Factor model (FFM) were labeled Agreeableness, Extraversion, Conscientiousness, Stability, and Openness to Experience. The Big Five (Goldberg, 1990) and the six-factor Hexaco (Lee & Ashton, 2008) models are instead based on factor analysis of personality terms found in dictionaries. This lexical approach to personality research is based on the *lexical hypothesis* that states that personality traits and differences that are the most important and relevant to people are described in natural language using words (Goldberg, 1982; Klages, 1926). The five broad traits are essentially the same in the FFM and Big Five models, while the Hexaco model has Honesty-Humility as the sixth factor.

Many prominent personality tests, such as the NEO-PI-R (Costa & McCrae, 1992) and Hexaco-PI (Lee & Ashton, 2004) are based on the five- and six-factor models of personality. Extensive empirical evidence on the good predictive validity of these tests provides major support for the underlying theoretical models. However, attempts to find the neurophysiological correlates of the broad five or six factors have until now been unsuccessful (Saucier, 2018; Trofimova, Robbins, Sulis, & Uher, 2018), and without further proof the Big Five and the Hexaco models are to be understood simply as strongly abbreviated and abstract versions of the lay lexicon of personality terms (Asendorpf, 2016; Mottus, 2020; Revelle & Elleman, 2016).

However, even if the broad factors are, as suggested by Franić, Borsboom, Dolan, and Boomsma (2014) and Wiernik, Yarkoni, Giordano, and Raghavan (2020) mere statistical constructs emanating from factor analysis and not real psychological entities this does not falsify the entire lexical hypothesis. But what are the other options besides factor analysis for extracting the most important traits from the pool of lay descriptions? According to McCrae, Gaines, and Wellington (2012), “*the FFM arose precisely because the thousands of lay adjectives …were unmanageable*.” One alternative solution, based on cluster analysis, has been suggested by Wood, Nye, and Saucier (2010), who developed the Inventory on Individual Differences in the Lexicon (IIDL), which has 61 scales based on 61 clusters of three or more synonymous terms extracted from a pool of 504 frequently used personality descriptors. Another approach has been proposed by Roivainen (2016) based on the observation that the frequency distribution of personality terms roughly follows Zipf’s (1949) law, which states that in a corpus of words, the frequency of any word is inversely proportional to its rank in the frequency table. Therefore, if word frequency correlates even moderately positively with the importance of the corresponding trait, then a list of the most frequently used 40–50 terms (excluding synonyms and antonyms) most likely includes all significant traits. Roivainen’s studies (2013, 2015, 2016) also showed that those Big Five/Hexaco marker adjectives that represent the cores of the broad factors are not more frequently used words than are “blended adjectives” with moderate loadings on several factors. This means that laymen do not recognize the high loading marker adjectives as especially useful personality descriptors in everyday use. A third option to discover the most important traits in the lexicon is to study personality descriptions in more limited lexicons. For example, we may hypothesize that new immigrants to a country first learn those words that are most needed for everyday communication (“hello,” “thank you,” “good,” “bad”) and this also applies to personality descriptors. Expressions such as “good man” and “bad person” are probably learned before more advanced-level descriptions such as “agreeable man” and “conscientious person.” Another group of persons with limited vocabularies is children.

In the present study, the age of acquisition (AoA) of common personality adjectives was investigated as another potential indicator for important traits. The typical first words used by infants include *mama, dada, ball, hi*, and *dog*, and the average 1-year old child has a vocabulary in comprehension of roughly 100 words. At the age of 18 months, the average passive vocabulary is around 600 words and the average productive vocabulary is close to 100 words. Toward the age of 30 months, the total productive vocabulary increases to more than 1,000 words (Mayor & Plunkett, 2011). Studies on the AoA of words (Brysbaert & Ellis, 2016; Dale & O’Rourke, 1981) show that common personality descriptors are included in the vocabulary of 2–4 year olds. We may hypothesize that children first learn those personality-related concepts that are important in their lives: these words may describe the child herself (*don’t be shy!*), friends or family members (*be brave like your brother!*) or other significant characters (*Practical Pig is smarter than Zeke the Wolf, who is evil*). The child gradually acquires a theory of mind and personality (Brown, Mangelsdorf, Agathen, & Ho, 2008; Eder, 1990), and as the lexicon of psychological concepts becomes larger, these implicit models become more refined and sophisticated.

Early-acquired words are processed faster than words with a later AoA in semantic categorization tasks (Brysbaert, Van Wijnendaele & De Deyne, 2000), in lexical decision tasks (Cortese & Khanna, 2007), and in pictured object naming (Holmes & Ellis, 2006; Navarrete, Pastore, Valentini, & Peressotti, 2015). They also tend to be more positive, less dominant, more easily imageable and more frequently used as compared to words learned later on in life (Montefinese, Vinson, Vigliocco, & Ambrosini, 2019; Moors et al., 2013; Morrison & Ellis, 2000). However, according to Brysbaert and Biemiller (2017), words learned early in life are processed more efficiently than later-acquired words, even when word frequency, word length, and similarity to other words are controlled for. Early acquired concepts are richer, more accessible, and also, for example, more robust against brain damage (Brysbaert & Ellis, 2016). These effects have been explained by the semantic locus hypothesis that suggests that early-acquired words help to build the semantic network for the acquisition of later-learned words (Steyvers & Tenenbaum, 2005) and consequently they have more connections compared to later-acquired words.

The present study investigates the relationship between AoA, word frequency, and Big Five/Hexaco factor loading of personality adjectives. It is hypothesized that words that describe important traits are learned at an earlier age, have higher frequency of use, and have higher loadings in the factor models. A high (negative) correlation between AoA and factor loading in one factor model and a lower correlation with loading in another factor model may be interpreted as supporting the validity of the former model. A low correlation between AoA and factor loading for both the Big Five and Hexaco models may be interpreted as supporting other trait taxonomies.

## Method

Big Five factor loadings reported for 339 personality-descriptive adjectives by Saucier and Goldberg (1996) and Hexaco factor loadings reported for 449 adjectives by Lee and Ashton (2008) were used in the study. These terms originally come from Norman (1967), who created a list of 2,800 personality adjectives based on the study by Allport and Odbert (1936), who found 18,000 terms that describe personality or behavior in dictionaries. Norman excluded from Allport and Odbert’s list those words that were purely evaluative, obsolete, or described physical characteristics. The set of 2,800 adjectives was further cut down to 1,711 words by Goldberg (1990), who used a panel of 10 student judges to pick the most familiar terms on Norman’s list. Consecutively, larger panels of judges were used by Lee and Ashton and Goldberg to reduce the list of adjectives to 449 and 339 words, respectively.

The data for word frequency was adopted from the studies by Roivainen (2015, 2016), who investigated the frequency of the use of personality adjectives as qualifiers of the nouns *person* and *woman* (e.g., *intelligent person, kind woman*) in the Google Books corpus, which is the largest existing text corpus, comprising 155 billion words in 2012 (Michel et al., 2011). Word frequency for the year 2000 was reported in these studies using 3 year-smoothing (= mean word usage for the years 1997–2003). In the present study, frequency of the use of personality adjectives as qualifiers for the noun *boy* was additionally studied using the same methodology. Three different qualified nouns, *person, woman*, and *boy* were used as previous studies show that there are small differences in the usage frequencies of adjectives depending on the qualified noun. For example, in the study by Motschenbacher and Roivainen (2020), correlation between usage frequency for adjectives qualifying *woman* and *person* was *r* = .71, and between *man* and *person r* = .67. Word frequencies of adjectives qualifying *boy* were analyzed in the present study to control for the potential differences in the description of adults (*woman*) and children (*boy*).

The data on AoA of the words came from Brysbaert and Biemiller (2017) and is largely based on the study by Dale and O’Rourke (1981), who used three-alternative multiple choice test items to analyze the knowledge of 31,000 words in American children from school grades 4, 6, 8, 10 and 12 and college students (grades 13 and 16). Each word was tested with 200 children. The testing took place between the years 1950–1980 in the US Midwest. The criterion for AoA for a word was that 70–80% of children at a specific grade level knew the meaning of that word. The Dale and O’Rourke compilation was expanded by Biemiller (2010), who made an additional category for grade 2, in which he included the words known by more than 80% of the children in grade 4. Data from studies by Goodman, Dale, and Li (2008) and Morrison, Chappell, and Ellis (1997) that investigated vocabulary of toddlers and kindergarten-age children were additionally used for the AoA estimates by Brysbaert and Biemiller (2017).

The Brysbaert list gives different AoA ages for different meanings of a word. For example, AoA for *warm* meaning “medium hot” is grade 2 while the AoA for *warm* meaning “kindly” is grade 6. Obviously, in these cases, the AoA for the meaning involving personality description was used. Among the personality terms studied by Saucier and Goldberg (1996), there are many pairs of adjectives consisting of a frequently used term such as *intelligent* or *critical* and a less frequently used antonym formed by the prefix non- un-, or in-, for example, *uncritical* and *unintelligent*. While these pairs of terms are most likely learned at the same age, only those adjectives that appeared on the Brysbaert list were accepted for the present study. In all, AoA for 274 of the 339 adjectives used by Goldberg (1990) and AoA for 408 adjectives out of the 449 studied by Lee and Ashton (2008) were included in the analyses.

Desirability ratings (scale −3 to +3) of adjectives from the study by Chandler (2018) were compared to AoA data. In all, 250 adjectives out of the 408 analyzed in the present study had been rated in Chandler’s study.

The data set is freely available (see Supplementary Materials).

## Results

### Age of Acquisition of Big Five Adjectives

Table 1 shows 28 Big Five adjectives that are learned before or during second grade, another 33 adjectives that are learned before or during 4th grade, and 15 adjectives that are typically learned after the 12th grade. Thus, 198 of the Big Five adjectives analyzed in the present study are learned and become part of active vocabulary between the 5th and 12th grades.

**Table 1:** Early and Late-Acquired Personality Adjectives

Age of Acquisition				
Grade 2		Grade 4		Grade 13+
Bashful (E)	Pleasant (A)	Absentminded (C)	Insecure (S)	Condescending (A)
Brave (E)	Polite (A)	Active (E)	Jealous (S)	Cordial (A)
Bright (O)	Proud (E)	Artistic (O)	Mannerly (C)	Devious (A)
Careful (C)	Quiet (E)	Bossy (A)	Nervous (S)	Fretful (S)
Careless (C)	Shy (E)	Bull-headed (A)	Nosey (S)	Gregarious (E)
Cheerful (E)	Smart (O)	Carefree (E)	Patient (A)	Introspective (O)
Cruel (A)	Talkative (E)	Courageous (E)	Rude (A)	Pompous (A)
Dependable (C)	Thoughtless (A)	Crabby (A)	Selfish (A)	Vindictive (A)
Forgetful (C)	Trustful (A)	Cranky (A)	Shallow (O)	Docile (E)
Friendly (A)	Truthful (A)	Dishonest (A)	Silent (E)	Exacting (C)
Helpful (A)	Unkind (A)	Excitable (S)	Sloppy (C)	Impetuous (E)
Kind (A)		Explosive (A)	Sly (A)	Lethargic (E)
Lazy (C)		Grumpy (A)	Stubborn (A)	Self-indulgent (A)
Merry (E)		Honest (A)	Suspicious (A)	Surly (A)
Greedy (A)		Humorous (E)	Unfriendly (A)	Unassuming (A)
Peaceful (A)		Impolite (A)	Unreliable (C)	
Playful (E)		Industrious (C)		

*Note*. Age of Acquisition from Brysbaert and Biemiller (2017). Personality adjectives from Saucier and Goldberg (1996). Big Five factors (factor with primary loading in Saucier & Goldberg, 1996): E = Extraversion, A = Agreeableness, C = Conscientiousness, S = Stablity, O = Openness.

As Table 1 shows, only few adjectives known by 2nd—4th graders describe the Stability and Openness factors of personality. In the Big Five framework, 28 of the adjectives known by 2nd—4th graders have their highest loading on the Agreeableness factor, 14 describe Extraversion, and 10 are Conscientiousness adjectives. The correlation between AoA and Big Five primary loading was *r* = −.24, *p* < .001.

### Age of Acquisition of Hexaco Adjectives

Table 2 shows the number of adjectives at each level of AoA, the mean word frequency, and the mean primary factor loading of the adjectives in the Hexaco model. As Table 2 indicates, words that are learned at an early age have a higher frequency of use as compared to words learned at later stages. The correlations between AoA and word frequency for adjectives qualifying *person*, *boy,* and *woman* were *r* = −.19, *p* < .001, *r* = −.28, *p* < .001, and *r* = −.21, *p* < .001, respectively. The difference in word frequency between the 96 words known by grade 4 and the 80 words known only by the 12th and 14th graders categories is significant for adjectives qualifying *person*, *t* (174) = 2.95, *p* = .0036; *woman, t* (174) **=** 3.15, *p =* .0019; and *boy*, *t* (174) = 3.78, *p* < .001.

**Table 2:** Personality Adjectives: Age of Acquisition (AoA), Word Usage Frequency and Factor Loading

AoA before School Grade	Number of Terms	Number of Terms with Primary Loading on Hexaco Factor						Mean Word Frequency Qualifying			Mean Primary Loading
		x	c	a	o	e	h	*Person*	*Boy*	*Woman*	
2	39	12	10	5	1	5	6	20	6.2	18.1	41
4	57	8	11	17	2	11	8	14.6	1.7	9.7	36.4
6	96	16	25	19	7	19	10	12.9	1.4	9.9	37.5
8	90	15	17	13	12	12	21	9.3	0.9	5.6	38.6
10	46	9	9	9	8	5	6	5.2	0.4	3.6	34.9
12	57	14	9	5	15	4	10	7.7	0.4	4.4	36.0
14	23	3	6	1	7	3	3	4.1	0.3	2.6	31.0
*Total*	*408*	*77*	*87*	*69*	*52*	*59*	*64*				

*Note*. Age of Acquisition (AoA) from Brysbaert and Biemiller (2017). Google books Ngram word frequency per billion words. Primary factor loading from Lee and Ashton (2004). Hexaco factors: eXtraversion, Conscientiousness, Agreeableness, Openness, Emotionality, Honesty-Humility.

As Table 2 shows, adjectives in the Openness category seem to be learned at a later age than other adjectives. There were only four adjectives that had their highest loading on the Openness factor that are known to 2nd—4th graders: *bright*, *smart, artistic,* and *clever*. The correlation between mean primary loading in the Hexaco model and AoA was fairly low, (*r* = −.17, *p* < .001). However, the mean loading was slightly higher (41) for the youngest grade 2 category as compared to the older children (36.6); *t*(406) = 3.10, *p* = .0020.

Word desirability had a weak negative correlation with AoA, (*r* = −.10, *p* = .114). The difference observed between the mean desirability of adjectives learned before grade 2 (0.77) and that of adjectives learned in grade 4 or later (0.19) approached significance, *t* (248) = 1.94, *p* = .0530.

## Discussion

The results of the study show that children first learn those personality terms that have high usage frequency in books, and presumably in language in general. Words that describe the broad Openness and Stability/Emotionality factors of personality are learned somewhat later than words for the other broad factors. Young children (2nd Graders and younger) seem to learn adjectives that describe the core features of the Big Five and Hexaco domains slightly earlier than those adjectives that refer to more peripheral traits in these factor models. However, the correlations between AoA and factor loading were low when older children were included in the analysis. There are several potential explanations for this:

*Differences between adult and children’s personality*. The Big Five and Hexaco models are based on studies with adults and may not be fully valid for children and adolescents. Hampson and Edmonds (2018) conclude that the Big Five can be identified in young children, and children's Big Five traits can predict adult outcomes, even if the Big Five factor structure does not necessarily emerge as clearly as it does in adults. It has been suggested that early temperamental traits may develop into more complex personality traits as the child matures (Caspi, Roberts, & Shiner, 2005; Shiner & Caspi, 2003). For example, Agreeableness and Conscientiousness may reflect a common trait in childhood and become differentiated in older children (Rothbart, Ahadi, Hershey, & Fisher, 2001; Soto, John, Gosling, & Potter, 2008). The broad domain of Openness is also absent from early age temperamental models suggesting that this trait may emerge at later stages of childhood (Lamb, Chuang, Wessels, Broberg, & Hwang, 2002; Tackett et al., 2012). Alternatives to the Big Five and Hexaco taxonomies have been suggested, for example, by Wilson, Schalet, Hicks, and Zucker (2013) who found two broad personality dimensions in children and labeled them *adaptive socialization* and *anxious inhibition,* and by Soto (2016) who has proposed a six-factor model integrating personality and temperament theories for describing the personality of children and adolescents. The “little six” model by Soto includes Activity as the sixth broad factor in addition to the traditional Big Five factors. Such alternative models of childhood personality were not included in the present study, and it may be that the correlation between AoA and factor loading would be higher for these models than for AoA and Big Five/Hexaco models. For example, the observation that Openness-related adjectives are learned at a later age than other adjectives seems to conform to the hypothesis that this trait emerges in older children.

*Different functions of personality terms in language.* While the Big Five and Hexaco models aim to be maximally efficient for the purposes of differential psychology, the personality lexicon that children learn from their parents and friends has many other important functions involving social interaction. In the present study, personality adjectives that are learned before grade 2 were found to have both higher desirability ratings and higher usage frequency than words learned at a later age. This finding is in accordance with previous research, such as the study by Wood (2015), who found desirability and usage frequency to have a correlation of .25 in a sample of 504 common personality terms. According to Wood, “The association between a term’s relational impact and its frequency of use may in turn be mediated by normative pressures which cause the actual frequency of socially valued traits to increase.” Such factors may also partly explain why words such as *friendly* and *wise* are learned at a very young age, despite their being distant from the cores of the broad factors.

*Non-representative sample of adjectives*. The groups of adjectives studied in the present study are highly selected samples. The correlations between AoA and word frequency observed in the present study (between *r* = −.19 and *r* = −.28) were much lower than that observed by Brysbaert and Biemiller for the 18,000 words in their study, *r* = −.587. This could be explained by the selected nature of the sample of adjectives used in the present study. In Goldberg’s study, student panels were used to select the most familiar adjectives from the list of 2,800 adjectives created by Norman. Thus, the words studied by Brysbaert and Biemiller probably also include much more rarely used terms. The same logic applies to the observations involving factor loadings. The observed correlations (*r* = −.17 and *r* = −.24) were fairly low. However, if there were factor loading data available for 2,800 adjectives rather than only for the 300–400 most popular adjectives, it might be that the adjectives at the core of the five broad factors would have clearly lower AoAs as compared to adjectives at the margins of the broad factors.

*The Big Five and Hexaco factors are not real psychological entities*. In a recent discussion article (Open Peer Commentary, 2020) on the validity of the Hexaco model, many influential personality scientists seem to take this view. According to Saucier (2020), "The Big Five is an empirically derived structure that has tended (and still tends) to be theoretically ill‐defined." Wiernik et al. (2020) argue that, "Any attempt to carve personality space into a low‐dimensional (e.g., five‐ or six‐factor) solution necessarily discards important information," and call for "a moratorium on attempts to identify a universally optimal low‐dimensional personality structure." Mottus (2020) concludes that, "Choosing between any number broad domains (three? five? six?) will always be an arbitrary decision." In a similar vein, Revelle, Dvorak, and Condon (2020) believe that, "By forming higher level constructs and ignoring the meaningful signals available at the item level, our field has been led astray." Consider the trait of honesty that is a peripheral trait in the Big Five model, falling between the Agreeableness and Conscientiousness superfactors, while in the six-factor scheme, Honesty-humility is one of the broad factors. As Table 1 shows, *dependable, truthful, honest,* and *unreliable* are among personality descriptors that are learned at an early age. The frequency of the use of *honest* as qualifying *person* in book texts is roughly equal to the sum of the frequencies of the 100 terms with lowest usage frequencies used in the study by Saucier and Goldberg (1996). Thus, the AoA and frequency data suggest that honesty is an important trait. However, in factor analytic models, the status of this trait varies considerably depending on how many factors are extracted.

The paradigmatic factor analytic studies in personality science might also be criticized for having an inconsistent attitude toward lay theories of personality. While it is assumed that the lay lexicon reliably covers the semantic field of personality description, there is a lack of trust in the results of the evolution of language involving the division of the semantic field into words. Hierarchical taxonomies are highly common in natural language, for example, letter-word-language, computer-device-object, and writing-communicating-doing something. The absence of lay words that would correspond to the five or six broad personality domains in the factor models may be caused by shortcomings in observation and analysis of behavior by laypeople, but another possibility is that there are no real referent entities.

Attempts to find the neurophysiological correlates of the broad five or six factors have until now been unsuccessful (Trofimova et al., 2018). If the broad factors are not real psychological entities, the description of personality at the facet level should probably be independent of the broad factors, as proposed by Wood et al. (2010) and Saucier, Iurino, and Thalmayer (2020). Condon and Mroczek (2016) observe that "Hierarchically nested facets (like those for the NEO-PI-R)..., are intrinsically limited in scope to the multidimensional space of the highest level; they are a 30-factor model of the Big-Five space but not a 30-factor model of personality space." Thus, for a model with 30 facets, the semantic field of personality descriptors should be directly divided into 30 parts and not into five parts that are then divided into six parts each. Correlational studies involving the identification of clusters of personality terms, and the analysis of their frequency of use, desirability, and AoA are complementary methods for identifying those terms that are fundamental in personality description.

If the critics cited above are correct and the use of a larger number of narrow facets in the description of adult personality outperforms descriptions based on a small number of broad traits, then the usefulness of the Big Five and Hexaco models in developmental psychology is even more questionable. Studying the ontogeny of the 15, 21 and 28 personality dimensions identified by Saucier and Iurino (2019), that of the 61 traits found by Wood et al. (2010), or the 23 personality facets analyzed by Roivainen (2016) may be more fruitful for theories of childhood personality.

Arguably, the approach of classical theories such as those by Erikson (1980), Vygotsky (1987) or Piaget (1970) that describe and explain the development of narrow traits identified by laypersons, for example, shyness, creativity, and selfishness, is perfectly valid. Observations on the AoA of personality terms might be also used to analyze the validity of such classical theories. For example, do children learn words that denote aspects of being independent and self-reliant versus helpless and clinging at an especially fast rate between the ages of 3 to 5, a period that corresponds to Erikson’s third stage of development (Purpose, initiative vs. guilt)? Or, do children learn concepts referring to creativity (creative, imaginative, original) later than synonyms for intelligence (intelligent, rational, wise) and is there an association between word knowledge and manifest behavior such as suppressed creativity in primary-school aged children (Gardner, 2008; Vygotsky, 1987)? Moreover, how does the level of the abstractness of personality terms known by the child correspond to his/her interpersonal skills and theory of mind?

## Conclusions

Analysis of the AoA of personality terms represents a genetic method for the study of the individual personality lexicon and offers a potential alternative to correlational analysis for identifying the fundamental personality descriptors among the thousands of terms that appear in language. Young children seem to learn personality terms that represent traits at the cores of the broad personality factors in the Big Five and Hexaco models slightly earlier than words that represent traits that are more peripheral. This may be interpreted as supporting the notion that the broad factors are real psychological entities and not just statistical constructs. However, in older children beyond second grade, correlations between factor loading and AoA are weak. Words that describe the broad Openness and Stability factors of personality are learned somewhat later than words for the other broad factors. It is hypothesized that the AoA of a personality term reflects the importance of the corresponding trait for children and may be used as one criterion for selecting facet level scales for personality tests. Further analyses of AoA of personality terms are needed, as they may contribute to theories of childhood personality, the development of the theory of mind in children, and the development of personality tests.

## Supplementary Materials

## Supplementary Materials



RoivainenE.
 (2022). Supplementary materials to "Age of acquisition of personality terms: Implications for personality theory"
[Dataset]. PsychOpen. 10.23668/psycharchives.6528
PMC963255036348698

## Data Availability

For this study a data set is freely available (see Supplementary Materials).
